# Effects of Catalyst Synthesis Methods on the Physicochemical Properties of Silica‐Supported Au–Ru Bimetallic Catalysts and their Influence on the Oxidation of Phenols with H_2_O_2_

**DOI:** 10.1002/open.202400484

**Published:** 2025-04-10

**Authors:** Tumisang Lekgetho, Matshawandile Tukulula, Letlhogonolo Fortunate Mabena, Mabuatsela Virginia Maphoru

**Affiliations:** ^1^ Department of Chemistry Tshwane University of Technology Private Bag X680 Pretoria 0001 South Africa; ^2^ School of Chemistry and Physics University of KwaZulu‐Natal Private Bag X01 Durban 4000 South Africa

**Keywords:** binaphthones, oxidations, phenols, quinones, Ru–Au catalysts

## Abstract

Herein, silica‐supported Au–Ru catalysts with 5% loading for each metal were prepared by microwave‐assisted loading (MW) and deposition (DP) methods. Au–Ru nanoparticles are obtained on MW‐5Au5Ru while short Au–Ru nanochains are obtained on DP‐5Au5Ru. The performance of the catalysts is tested through the oxidation of 2,3,5‐trimethylhydroquinone (**TMHQ**) and 4‐methoxy‐1‐naphthol (**MNL**) with H_2_O_2_, in which 2,3,5‐trimethyl‐1,4‐benzoquinone (**TMBQ**) and 4,4′‐dimethoxy‐2,2′‐binaphthalenylidene‐1,1′‐dione (**BNP**) are produced as main products, respectively. Catalytic data obtained for the oxidation of **TMHQ** demonstrate that the structures of the catalysts, type of solvent, and reaction temperatures used have a significant influence on the activities and selectivities of the catalysts. When MeOH and MeNO_2_ are used at room temperature (RT) in the oxidation of **TMHQ** on MW‐5Au5Ru catalyst, 58.2% and 100% conversions of **TMHQ** are achieved, respectively. Both MW and DP‐synthesized catalysts are highly active in the oxidation of **TMHQ**. Similar to **TMHQ**, the catalytic outcomes on the oxidative coupling of **MNL** highly depend on the temperature and structure of the catalyst. For example, 34% and 96% conversions of **MNL** are achieved at RT and 60 °C, respectively, over MW‐5Au5Ru catalyst in MeOH. However, **MNL** conversion of 82% is achieved on DP‐5Au5Ru catalyst in MeOH at RT.

## Graphical abstract

The catalytic oxidative conversion of 2,3,5‐trimethylhydroquinone and 4‐methoxy‐1‐naphthol using gold–ruthenium catalysts supported on SiO_2_ results in the formation of varying quantities of 2,3,5‐trimethyl‐1,4‐benzoquinone and 4,4’‐dimethoxy‐2,2’‐binaphthalenylidene‐1,1’‐dione, respectively. The catalysts, which are synthesized using microwave‐assisted loading and deposition techniques, exhibit varying morphological characteristics, which have a high impact on the product yields and selectivities.

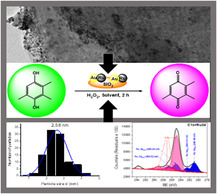

## 1 Introduction

**Figure 1:**
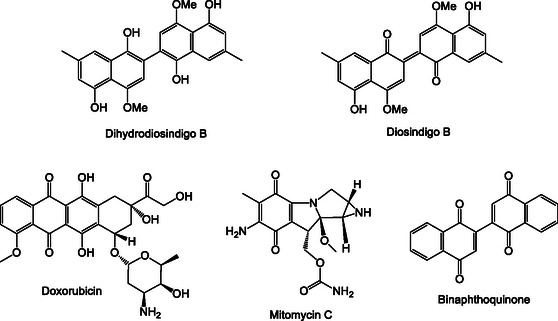
Examples of natural binaphthols, binaphthones, quinones, and binaphthoquinones.

Hydroxyarenes are key substrates in the synthesis of an array of biologically active organic compounds.^[^ ^1^, ^2^ ^]^ Their chemical reactions are essential in the fabrication of biaryl and triaryl aromatic structures, which are central building blocks for many synthetic and natural macromolecules.^[^ ^3^ ^]^ Quinones, for example, are fundamental building blocks for a wide variety of biologically active compounds and a vast family of vitamins that are often used as antioxidants.^[^ ^4^, ^5^ ^]^ The reactions of hydroxyarene derivatives, such as phenols and binaphthols, are known to yield monoquinones, binaphthoquinones, binaphthols, and binaphtho‐furans.^[^ ^6^, ^7^ ^]^ Naphthols derivatives serve as key intermediates in the synthesis of biologically active synthetic and natural products such as dihydrodiosindigo B and diosindigo B (**Figure** **1**).^[^ ^8^ ^]^ Diosindigo B is a naturally existing binaphthone often extracted from the heartwood of Diospyros melanoxylon and used as a herbal remedy for the treatment of whooping cough, menstrual problems, and abdominal pains.^[^ ^9^ ^]^ Doxorubicin and mitomycin C are natural and synthetic compounds that can be isolated from Streptomyces peucetius and Streptomyces caespitosus, respectively.^[^ ^10^ ^]^ They are used for the treatment of breast, bladder and lung cancers, bone and soft tissue sarcomas. Moreover, they are employed in the treatment of ovary and acute lymphocytic leukemia and thyroid carcinoma, among other illnesses.^[^ ^11^, ^12^ ^]^ Some binaphthoquinones are used for the treatment of Candida albicans due to their antifungal activities.^[^ ^13^ ^]^

Transition metals such as Ag(I), Cr(IV), Cu(II), Fe(III), Mn(III), and Pb(IV) were often used in their stoichiometric amounts in the oxidation of hydroxyarenes.^[^ ^14^ ^]^ One typical example is the oxidation of phenols to 1,4‐benzoquinones using stoichiometric amounts of copper(I) chloride alongside O_2_.^[^ ^15^ ^]^ The authors reported low substrate conversions and product selectivities for the reactions of mono‐ and unsubstituted phenols.^[^ ^15^ ^]^ Kholdeeva and co‐workers also conducted the oxidative transformation of 2‐methyl‐1‐naphthol with O_2_ as an oxidant.^[^ ^16^ ^]^ The authors obtained low‐to‐moderate yields of menadione due to the presence of several side products that lowered the selectivity of the target product. Separation of major products from the byproducts remains one of the major problems since it increases the production costs of these typical reactions. Furthermore, handling the hazardous reduced inorganic oxidants from stoichiometric oxidation reactions of phenolic compounds is one of the major environmental challenges.^[^ ^17^, ^18^, ^19^ ^]^ Strategic transformation of these reactions using green methods that align with the United Nations‐Sustainable Development Goals is one of the major goals in the production of value‐added chemical compounds.^[^ ^20^ ^]^

The use of metal oxides and supported metals as catalysts for the oxidation of hydroxyarenes has been reported by other researchers over the past years.^[^ ^21^, ^22^ ^]^ Kholdeeva and co‐workers studied Au/C and Au/TiO_2_‐catalyzed oxidation of 2‐methyl‐1‐naphthol with O_2_ as an oxidant.^[^ ^23^ ^]^ They achieved 2‐methyl‐1‐naphthol conversions that range between 79% and 94% given the reaction conditions used.^[^ ^23^ ^]^ Jawale and co‐workers also obtained exceptional yields of the targeted 1,4‐benzoquinones in the oxidation of 1,4‐dihydroxybenzene derivatives using AuCNT (CNT: carbon nanotubes) in the presence of NaHCO_3_ and K_2_CO_3_ bases.^[^ ^7^ ^]^ Previously, our research group reported on the coupling of 4‐methoxy‐1‐naphthol (**MNL**) and 2‐methyl‐1‐naphthol over Bi‐ and Sb‐promoted Pt/AC (AC: activate carbon) catalysts in various solvents using H_2_O_2_ as an oxidant.^[^ ^24^, ^25^, ^26^ ^]^ It was observed that the structures of the catalysts, which were influenced by their synthesis method, have detrimental effects on their catalytic outcomes.^[^ ^24^, ^25^, ^26^ ^]^ Furthermore, the reaction conditions, such as temperature and solvent, greatly impacted the outcomes of the catalytic reactions.^[^ ^24^, ^25^, ^26^ ^]^ In addition, the monometallic 5%Pt/AC catalyst contained agglomerated particles in comparison to dispersed metal NPs obtained on the bismuth‐promoted catalyst.^[^ ^25^ ^]^ This resulted in **MNL** conversion of 33.1% from 5%Pt/AC relative to 54.1% conversion obtained from 5%Pt‐5%Bi/AC under the same reaction conditions.^[^ ^24^ ^]^ This shows the importance of promoters on the overall activity of the catalyst and also on the structural properties and stability of the catalyst. Recently, our group reported on the oxidation of **TMHQ** on 5%Ru‐5%Bi/AC catalysts with various metal loadings.^[^ ^26^ ^]^ It was established, apart from metal loading, that the solvent and structural properties of the catalysts have an impact on the outcome of the catalytic reaction due to the interaction of the metals on the catalysts.^[^ ^26^ ^]^ A yield of 86.5% of **TMBQ** was obtained in MeNO_2_ at room temperature (RT) from deposition (DP)‐prepared 5%Ru‐5%Bi/SiO_2_ catalyst relative to 98.6% yield obtained from microwave (MW)‐synthesized 5%Ru–5%Bi/SiO_2_ catalyst. It should be noted that the noble metals are active metal catalysts in these reactions and Bi serves as a promoter.^[^ ^22^, ^24^, ^25^, ^27^ ^]^ The use of noble metal alloys and bimetallic catalysts has been applied in many catalytic oxidation systems and found to be highly active due to the synergistic effects between the noble metals.^[^ ^25^, ^28^, ^29^, ^30^ ^]^ This study examines the influence of catalyst fabrication methods, MW‐assisted loading, and DP method, on the morphologies of silica‐supported 5Au5Ru catalysts. The influence of their morphologies on their catalytic outcome in the oxidation of **TMHQ** and **MNL** will be determined. Furthermore, the impact of solvents and reaction temperatures on substrate conversion, product yields, and selectivities will be thoroughly discussed in this article.

## 2 Results and Discussion

### 2.1 Catalyst Characterization

**Figure 2:**
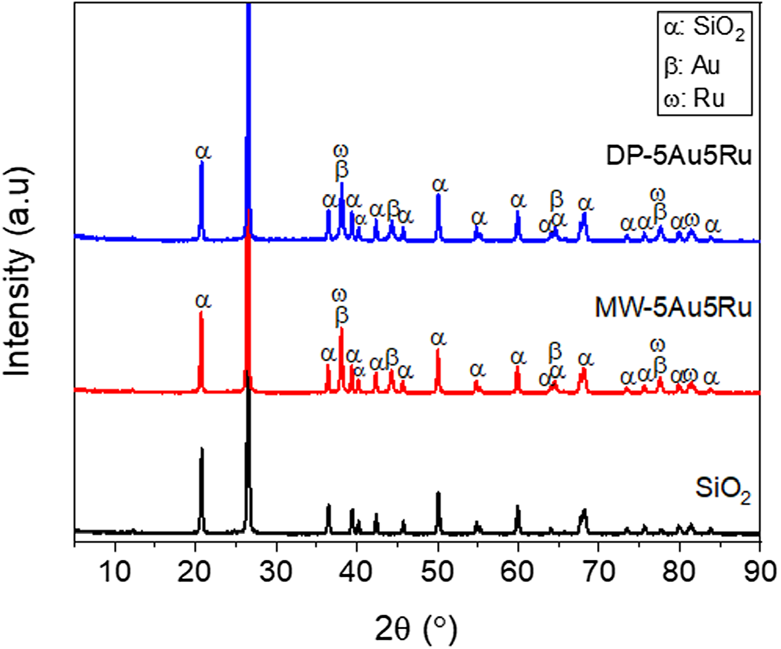
XRD patterns of SiO_2_,^[^ ^26^ ^]^ MW‐5Au5Ru, and DP‐5Au5Ru.

MW‐5Ru5Au and DP‐5Ru5Au catalysts were prepared by MW‐assisted loading and DP methods (Supporting Material 1, Supporting Information). The samples were characterized with X‐Ray diffraction (XRD), Brunauer–Emmett–Teller (BET) nitrogen physisorption, scanning electron microscopy (SEM), transmission electron microscopy (TEM), energy‐dispersive X‐Ray (EDX), X‐ray photoelectron spectroscopy (XPS), and inductively coupled plasma–optical emission spectroscopy (ICP‐OES) (Figure S2, Supporting Information). The XRD patterns for silica and silica‐supported catalysts shown in **Figure** **2** have diffraction peaks at 2θ = 20.64, 26.41, 36.35, 39.27, 40.69, 42.24, 45.59, 49.94, 54.71, 59.79, and 68.01°, which correspond to the (100), (101), (110), (012), (111), (200), (201), (134), (022), (211), and (122) lattice planes of crystalline hexagonal α‐SiO_2_ (quartz) support, respectively (COD 9 005 022). The XRD pattern of MW‐5Au5Ru possesses diffraction peaks at 2θ = 38.11, 44.32, 64.52, 77.52, and 81.63°, which represent (111), (200), (220), (311), and (222) lattice planes of face‐centered‐cubic (FCC) Au phase (COD 1 011 175), respectively. XRD peak reflections of Ru at 2θ = 38.30 and 77.55° on MW‐5Au5Ru are assigned to the hexagonal phase Ru (COD 1 539 052). Similar to the MW‐5Au5Ru, the XRD pattern of DP‐5Au5Ru in Figure 2 showed that it contains FCC and hexagonal phases of Au and Ru, respectively. There are no new peaks or shift in the diffraction patterns of either Ru or Au metals, which implies that the synthesized MW‐ and DP‐5Au5Ru is truly bimetallic.

**Figure 3:**
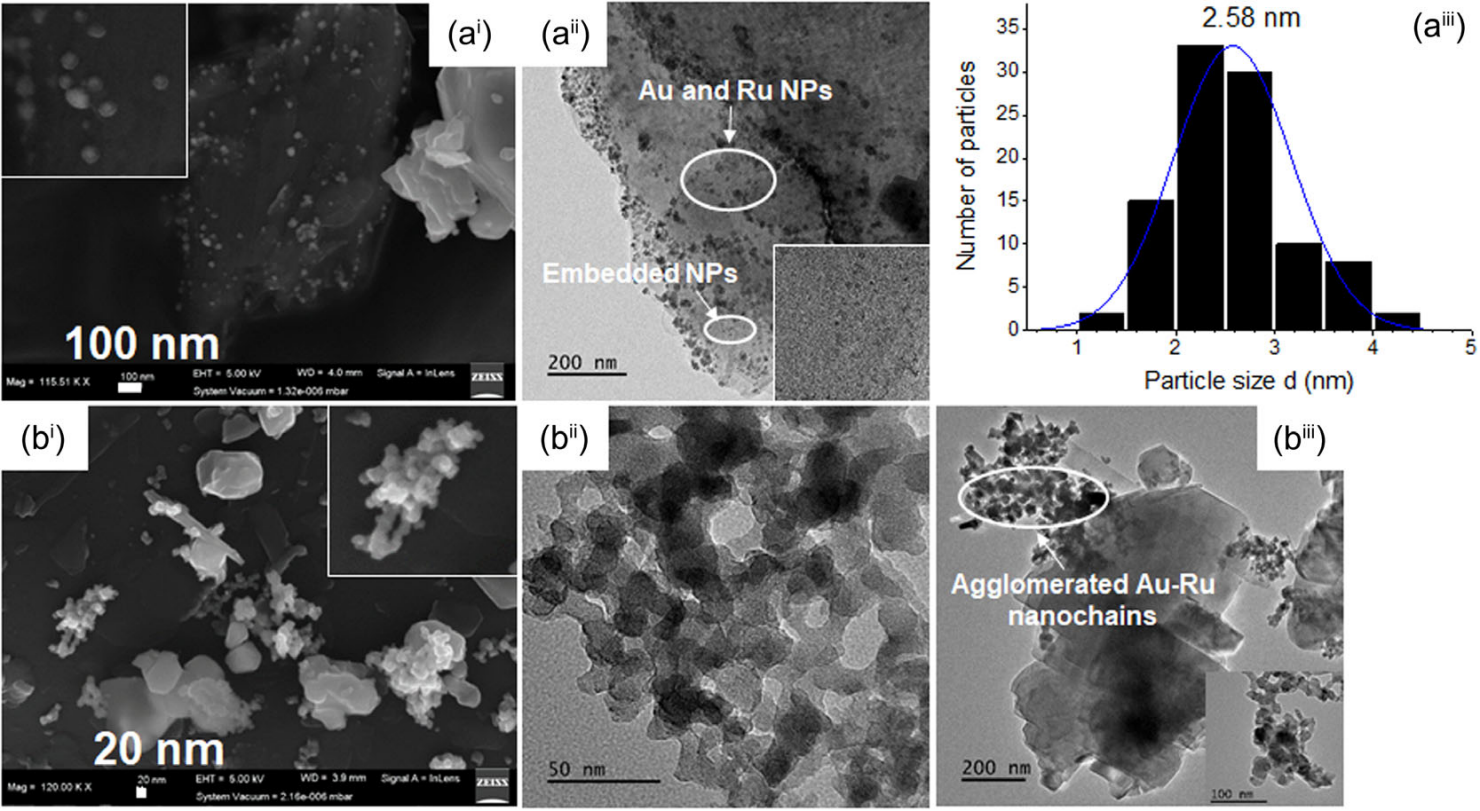
SEM micrographs, TEM micrographs, and PSD curve of a) MW‐5Au5Ru and b) DP‐5Au5Ru catalysts (PSD of DP‐5Au5Ru cannot be calculated for DP‐5Au5Ru due to its nanochain morphology).

Nitrogen physisorption data and metal loadings for SiO_2_ and SiO_2_‐supported 5Au5Ru catalysts prepared by MW‐assisted loading and DP methods are given in **Table** **1**. The BET SA (Surface area) of the commercial SiO_2_ support was found to be 6.6 m^2^ g^−1^. There was no significant change observed on the surface area of MW‐5Au5Ru from the support, but its average pore diameters (APD) increased slightly after metal loading. This is probably due to the blockage of the smaller pores by metal NPs, which are most likely to target the small pores and leaving large pores unoccupied. The SEM and TEM analysis of this catalyst in **Figure** **3**a shows that it contains small metal NPs with the average particle size (APS) of 2.58 nm. Contradictory to MW‐5Au5Ru, an increase in the BET SA was observed on DP‐5Au5Ru relative to the SiO_2_ support. The considerable increase in the BET SA on this catalyst may be due to the presence of the agglomerated nanochains that are isolated from the support, which may be adsorbing nitrogen (see SEM and TEM results in Figure 3b). Jin and co‐workers prepared Pd–Ni chains and obtained the surface areas of 39.2, 37.6, 67.1, and 72.3 m^2^ g^−1^ for Pd–Ni chains with molar ratios of 1:3, 2:3, 1:1, and 3:1, respectively.^[^ ^31^ ^]^ On the DP‐5Au5Ru, the surface area contribution from the chains is very low due to low metal loading on this catalyst. In addition, the catalyst fabrication method has the potential to increase the BET SA of the catalyst relative to that of the support due to the restructuring or formation of amorphous silica on the support surface during the catalyst preparation step.^[^ ^32^ ^]^ This was also observed on the MW‐prepared Ru‐Bi/SiO_2_ catalysts previously reported by us.^[^ ^26^ ^]^ Metal loadings on both catalysts are close to the target value of 5% for each of the metals (Table 1).

**Table 1:** Nitrogen physisorption data of silica and silica‐supported 5Au5Ru catalysts (drying temperature: 120 °C, drying time: 24 h).

Catalyst	BET SA	[t]‐Plot [S]	BJH [S]	PV	APD	Ru	Au
	[m^2^ g^−1^]	[m^2^ g^−1^]	[m^2^ g^−1^]	[cm^3^ g^−1^]	[nm]	[mass%]	
Silica	6.6	0.55	5.1	0.03	20.6	–	–
MW‐5Au5Ru	6.4	0.29	4.4	0.03	24.9	4.5	4.6
DP‐5Au5Ru	10.2	0.23	8.6	0.05	22.8	4.8	4.8

**Figure 4:**
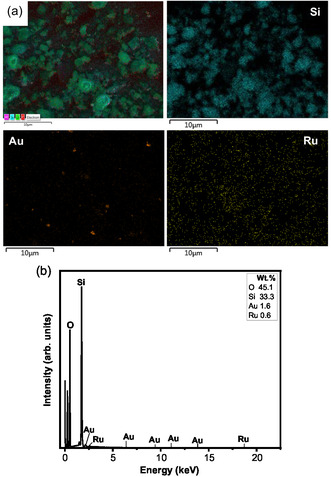
a) EDX imaging and b) EDX spectrum of MW‐5Au5Ru catalyst.

**Figure 5:**
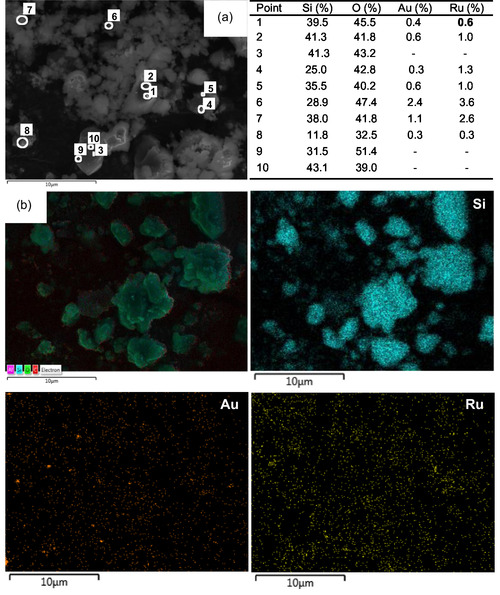
a) EDX “point‐and‐shoot” and b) EDX imaging of DP‐5Au5Ru catalyst.

The SEM micrographs of the MW‐5Au5Ru catalyst in Figure 3a ^i^ show the presence of spherical Au and Ru NPs that are dispersed on the surface of the support. Its TEM images further confirmed this observation (Figure 3a ^ii^). The APS of Au–Ru NPs on MW‐5Au5Ru was found to be 2.58 nm with a narrow particle size distribution (PSD) that ranges between 1.3 and 4.2 nm (Figure 3a ^iii^). The presence of both Au and Ru was confirmed by SEM–EDX imaging (**Figure** **4**b and Figure S3, Supporting Information**)** and “point and shoot” analysis (Figure S4, Supporting Information). EDX imaging analysis (Figure 4a) showed that Ru dispersed on the surface of the support with agglomerated Au particles (Figure 3a ^ii^). The Au agglomerated particles were also observed on its TEM image (Figure 3a ^ii^). Contrary to what was observed on the SEM images of the MW‐5Au5Ru, DP‐5Au5Ru (Figure 3b ^i^) contains mostly isolated nanochains that consist of clusters of interlaced Au–Ru particles on the catalyst surface. The short nanochains containing Au and Ru species were also confirmed by TEM analysis in Figure 3b ^ii^,b^iii^. It is clear from the TEM images that some of the nanochains are completely isolated from the support materials and they are likely to adsorb nitrogen during the nitrogen physisorption analysis. This explains the slight increase in the BET SA obtained on DP‐5Au5Ru relative to the support (Table 1). The EDX “point and shoot” and imaging analysis in **Figure** **5**a,b and Figure S5, Supporting Information) confirmed that the nanochains contain both Au and Ru species. The striking morphological differences between MW‐ and DP‐5Au5Ru catalysts demonstrate the impact of catalyst preparation methods on the final structure of the catalysts. Therefore, it can be concluded that MW preparation method promoted high metal dispersion than DP method. The formation of nanochains was also observed on 5%Pt‐5%Sb/AC and 5%Ru‐5%Bi/SiO_2_ catalysts that were prepared by DP methods, while highly dispersed metal nanoparticles were obtained on MW‐synthesized 5%Pt‐5%Sb/AC catalyst and less‐prominent Ru–Pd nanochains or agglomerated particles were obtained on 5%Ru‐5%Bi/SiO_2_.^[^ ^25^, ^26^ ^]^ Catalysts preparation by conventional DP methods are known to support the agglomeration of particles while MW‐based catalyst preparation methods are known to increase metal dispersion.^[^ ^25^, ^33^, ^34^, ^35^ ^]^

**Figure 6:**
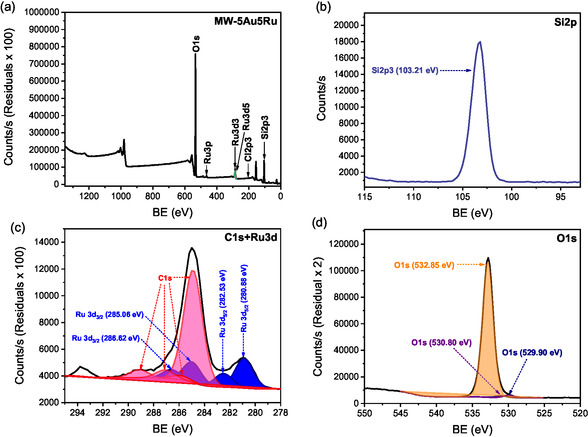
a) XPS survey spectrum and high‐resolution spectra of b) Si 2p, c) Ru 3d, C 1s, and d) O 1s for MW‐5Au5Ru.

**Figure 7:**
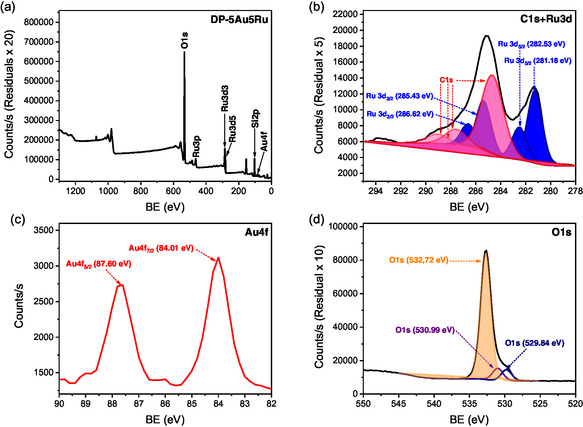
a) XPS survey spectrum and high‐resolution spectra of b) Ru 3d, C 1s c) Au 4f, and d) O 1s for DP‐5Au5Ru.

The XPS survey spectrum of MW‐5Au5Ru in **Figure** **6**a complements the EDX analysis in Figure 4. The prominent signal at binding energy (BE) of 103.21 eV is characteristic of Si 2p electrons of silica, which validates the existence of the silicon backbone in the catalyst support **(**Figure 6b). The BEs of 280.88 and 282.53 eV for two deconvoluted Ru 3d_5/2_ peaks were detected for two distinct Ru species (Figure 6c). Ru BEs between 280.9 and 281.2 eV are characteristic of RuO_2_,^[^ ^36^ ^]^ while weak peaks at higher BEs of 282.3–282.5 eV are characteristic of oxides of Ru in a higher oxidation state such as RuO_3_.^[^ ^37^, ^38^ ^]^ The signals with BE around 285.0–285.4 eV and 286.4–286.6 eV are characteristic of Ru 3d_3/2_ electrons in RuO_2_ and RuO_3_, respectively. The XRD patterns of this catalyst in Figure 2 confirmed the hexagonal phases of the reduced Ru metal present. This implies that the bulk sample contains metallic Ru with Ru metal oxides on its surface. It is widely known that metallic Ru oxidizes readily on its surface, even at RT, to form stable Ru oxide species.^[^ ^39^ ^]^ Hence, the observation made in Figure 6c is common. The low intensity doublet peaks were observed at BEs of 463.54 and 486.06 eV for Ru 3p_3/2_ and Ru 3p_1/2_, respectively. They indicate the presence of oxidized Ru species, RuO_2_, on the catalyst surface.^[^ ^40^ ^]^ The C 1s peaks on the XPS spectrum of MW‐5Au5Ru are due to carbon that originates from organic solvents, often present in high quantities during the catalyst synthesis process. In this case, the source of carbon may be coming from acetone used during the catalyst synthesis process or other carbon contaminants from the air. There are no peaks observed for Au on the XPS survey spectra of the MW‐5Au5Ru catalyst. The difficulty in detecting Au by XPS may be due to its poor dispersion (Figure 6a) and uneven distribution of its particles over the catalyst surface as shown on its TEM and EDX analysis (Figure 3a ^ii^,4 and Figure S3, Supporting Information). The detection of an oxygen signal at 529.90 eV confirms the existence of silica and supported metal oxides on this catalyst (Figure 6d). Similar to MW‐5Au5Ru, the same Ru metal and oxides species were observed on DP‐5Au5Ru catalyst. The XPS spectrum of Au 4f for the DP‐5Au5Ru catalyst shows double bands at 83.9 and 87.6 eV BE for 4f_7/2_ and 4f_5/2_ electrons (**Figure** **7**c) that correspond to the BE of pure bulk Au.^[^ ^41^ ^]^ This indicates that the electronic interactions between SiO_2_ and Au are weak since the BE of 4f_7/2_ is close to that of bulk gold metal.^[^ ^42^ ^]^

### 2.2 Oxidation of 2,3,5‐Trimethylhydroquinone and 4‐Methoxy‐1‐Naphthol

**Scheme 1:**
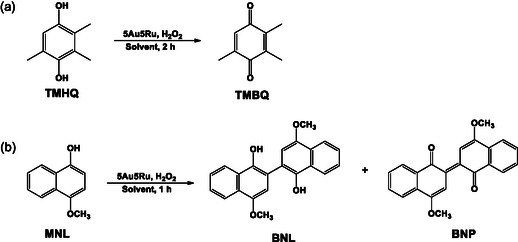
Oxidation of a) **TMHQ** and b) **MNL** over SiO_2_‐supported 5Au5Ru catalysts.

**Figure 8:**
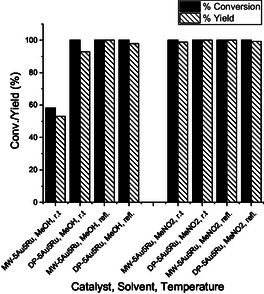
Conversions and yields obtained for the oxidative transformation of **TMHQ** to **TMBQ** on 5Au5Ru catalysts in different solvents and under different reaction temperatures (refl.: reflux).

**Figure 9:**
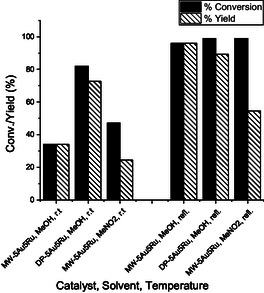
Conversions and yields obtained for the catalytic oxidation of **MNL** to **BNP** over silica‐supported 5Au5Ru catalysts in different solvents and under different reaction temperatures.

The substrates, 2,3,5‐trimethylhydroquinone (**TMHQ**) and 4‐methoxy‐1‐naphthol (**MNL**), were oxidized with H_2_O_2_ over MW‐ and DP‐5Au5Ru catalysts (**Scheme** **1**) according to the procedure described in Figure S6, Supporting Information.^[^ ^26^ ^]^ Product obtained for the oxidation of **TMHQ** is 2,3,5‐trimethyl‐1,4‐benzoquinone (**TMBQ**) while **MNL** gave 4,4′‐dimethoxy‐2,2′‐binaphthalenylidene‐1,1′‐dione (**BNP**). The reactions were carried out in either MeOH or MeNO_2_ at RT and/or in refluxing solvent. The conversions, selectivities, and yields obtained in these reactions are reported in **Table** **2**, **Figure** **8** and **9**.

**Table 2:** Oxidation of **TMHQ** and **MNL** on SiO_2_‐supported 5Au5Ru catalysts in MeOH or MeNO_2_.

Entry	Catalyst	substrate	Solv.	T [°C]	t [h]	Conv. [%]	Sel. [%]	
							**TMBQ**	**BNP**
1^a)^	MW‐5Au5Ru	TMHQ	MeOH	r.t	2	58.2	91.3	
2^a,b)^	DP‐5Au5Ru	TMHQ	MeOH	r.t	2	100	92.9	
3^c)^	MW‐5Au5Ru	TMHQ	MeOH	60	3	100	100	
4^a)^	DP‐5Au5Ru	TMHQ	MeOH	60	2	100	97.9	
5^a)^	MW‐5Au5Ru	TMHQ	MeNO_2_	r.t	2	100	98.9	
6	DP‐5Au5Ru	TMHQ	MeNO_2_	r.t	2	100	100	
7	MW‐5Au5Ru	TMHQ	MeNO_2_	96	2	100	100	
8^a)^	DP‐5Au5Ru	TMHQ	MeNO_2_	96	2	100	99.3	
9	MW‐5Au5Ru (used)	TMHQ	MeNO_2_	96	2	100	100	
10	MW‐5Au5Ru	MNL	MeOH	r.t	2	34.0		100
11^d,e)^	DP‐5Au5Ru	MNL	MeOH	r.t	1	82		88.7
12	MW‐5Au5Ru	MNL	MeOH	60	1	96		100
13^e)^	DP‐5Au5Ru	MNL	MeOH	60	1	≤99.0		90.2
14^d,^ ^e,f)^	MW‐5Au5Ru	MNL	MeNO_2_	r.t	1	47.1		52.0
15^c,^ ^d,e)^	MW‐5Au5Ru	MNL	MeNO_2_	96	1	≤99.0		55.0

a) A brown polymeric compound was identified (^13^C NMR);^[^ ^26^ ^]^

b) A purple minor product was observed;

c) Conversion of **MNL** after 2 h was very low;

d) Multiple minor byproducts with comparable retention factors were not separated by column chromatography;

e) Traces of a brown product were obtained;

f) Trace amounts of **BNL** (binaphthol) was formed.

Exceptional catalytic activities were obtained when **TMHQ** was oxidized on either MW‐ or DP‐5Au5Ru catalyst. A complete conversion of the **TMHQ** on both catalysts was obtained (Table 2, entries 2‐8) except for MW‐5Au5Ru in MeOH at RT, in which only 58.2% of **TMHQ** was converted to **TMBQ** (Table 2, entry 2). Both catalysts gave relatively comparable yields and selectivities for **TMBQ** (Table 2, Figure 8). Despite its high selectivity for **TMBQ**, MW‐5Au5Ru yielded a low amount of **TMBQ** due to the partial conversion of **TMHQ** (Table 2, entry 1 and Figure 8). The DP‐5Au5Ru catalyst is prone to minimal side reactions that led to the formation of multiple side products in comparison to MW‐5Au5Ru (Table 2, entries 2, 4 and 8). The emergence of byproducts in the reaction carried out on DP‐5Au5Bi led to a slight reduction of **TMBQ** selectivity on DP‐5Au5Ru. The SEM and TEM images of this catalyst showed that some of the Ru–Au nanochains, which are the active species, are not in contact with the support (Figure 3b). This surface heterogeneity may lead to diverse active sites with a wide spectrum of activities.

In the oxidation of **MNL**, both MW and DP‐5Au5Ru catalysts in MeOH at RT and at 60 °C always gave **BNP** as the major product (Table 2, entries 10‐15, Figure 9). Although high selectivities of **BNP** were attained on MW‐5Au5Bi catalyst, high **MNL** conversions were obtained on DP‐5Au5Ru catalyst in MeOH (Figure 9). The same trend was also observed for the oxidation of **TMHQ** (Figure 8). The selectivity in the oxidation reactions of **MNL** is affected by the multiple side products emerging from parallel reactions, which results in poor product selectivity. Maphoru and co‐workers also observed the formation of side products in the oxidative coupling of **MNL** with hydrogen peroxide over 5%Pt–5%Bi/AC catalyst.^[^ ^32^ ^]^

It is evident from the results obtained for the oxidation of **TMHQ** and **MNL** on MW‐ and DP‐5Au5Ru that the structure of the catalyst has a solid influence on the outcome of the reaction, especially in the oxidation of **MNL**. Between the two catalysts prepared by MW and DP methods, DP‐5Au5Ru catalyst proved to be the most active catalyst in comparison to MW‐5Au5Ru (Table 2, entries 2, 6 and 11). However, the MW‐5Au5Ru catalyst is more selective compared to the most active DP‐5Au5Ru catalyst. The selectivity on DP‐5Au5Ru was lowered by the formation of multiple side products on the catalyst surface. The occurrence of side reactions from the most active catalyst is quite common in these typical reactions.^[^ ^6^, ^24^ ^]^ In the study conducted on the oxidative coupling of 2‐methyl‐1‐naphthol on 5%Pt‐5%Bi/AC, it was established that the association of Pt and Bi has a direct impact of the outcome of the reaction. 5%Pt‐5%Bi/AC with associated metal were highly active due to the electronic effects and synergistic effects between the two metals.^[^ ^25^ ^]^ In this study, both catalysts contain Au and Ru, which are active metal catalysts in these reactions, but with different structural properties. DP‐5Au5Ru contains Au and Ru metals that are associated in the nanochains (Figure 3, 4, 5). Therefore, electronic and synergistic effects are at play between Ru and Au, which may be responsible for the high **MNL** conversions on this catalyst. This may be through an increase in the electron density on Au from Ru. However, a highly active catalyst is often prone to side reactions, as it was observed on DP‐5Au5Ru (Table 2). In addition, XPS and XRD analysis showed that there is a presence of oxidized Ru species on the catalyst surface with Ru metal in the bulk sample **(**Figure 2 and 7b **)**. Different metal species may possess different catalytic activities and routes, which are expected to reduce the selectivity of the reaction. Multiple reaction routes on the mechanism that lead to formation of monoquinones, binaphthofurans, and polymeric compounds are very common in the reactions of phenolic compounds.^[^ ^6^, ^43^, ^44^, ^45^, ^46^, ^47^ ^]^ In contrast to DP‐5Au5Ru, some Au agglomerates were dissociated from Ru NPs on MW‐5Au5Ru, which might have reduced the activity of the catalyst. Furthermore, Au is not very effective in dissociating hydrogen peroxide to peroxy and hydroxy radicals that are required for the oxidation of phenoxy and naphthoxy radicals.^[^ ^22^, ^37^ ^]^ Similar to the dissociation of hydrogen peroxide, it was previously proposed that the coupling of **MNL** occurs when the active metal catalyst cleaves the hydroxy group of the naphthol by abstracting its hydrogen.^[^ ^22^ ^]^ This is followed by the stabilization of the naphthoxy radicals by chemisorption on the metal surface. The ability of the metal to cleave and chemisorp the intermediates phenoxy and naphthoxy radical species is key to the activity and selectivity of the reaction. Therefore, partial isolation of Au from the ruthenium particles in this catalyst may be one of the contributing factors towards its low catalytic activities since each metal will function on its own. This may, in general, affect the kinetics and also the reaction route. Most of the oxidation reactions of the phenolic compounds follow this reaction mechanism.^[^ ^6^, ^43^, ^44^, ^45^ ^]^

Solvents have a significant influence in the oxidation of **TMHQ** on both catalysts. It was established that MeOH often favors the formation of side products than MeNO_2_ (Table 1, entries 1‐8). Overall, conversions, yields, and selectivities obtained for this reaction are relatively comparable for both solvents, even though MeNO_2_ is slightly better than MeOH. It can be concluded that MeNO_2_ is the best solvent for oxidation reactions of **TMHQ** since it offers complete conversions with selectivities of over 99% (Table 2) on both 5Au5Ru catalysts at RTand under reflux. MeOH has a higher donor number and polarity than MeNO_2_.^[^ ^48^, ^49^ ^]^ The donor number (DN) is used to determine the basicity of the solvent or its ability to donate electrons.^[^ ^50^, ^51^ ^]^ MeOH has a DN of 19.0 kcal mol^−1^, which is significantly higher that the DN of 2.7 kcal mol^−1^ for MeNO_2_. Oxidation of phenolic compounds often takes place in basic conditions. This was demonstrated by Jawale and co‐workers in the oxidation of 1,4‐dihydroxybenzene using AuCNT catalysts.^[^ ^7^ ^]^ Solvents with high donor numbers are expected to increase the basicity of the reaction medium, which promotes the oxidation of hydroxyarenes.^[^ ^7^, ^38^ ^]^ Furthermore, solvents with high polarity are known to stabilize the intermediates in the oxidation reactions and therefore facilitate the formation of the products.^[^ ^49^ ^]^ However, highly oxidative environments promote the formation of byproducts from side reactions, overoxidation, and multiple coupling reactions, which result in product selectivity reduction.^[^ ^6^, ^25^ ^]^ A similar trend was noted in the oxidation of **MNL** when reactions were conducted under identical conditions (Table 2, compare entries 10 and 12 to 13 and 14). Although low conversions were observed at RT over MW‐5Au5Ru catalyst in both MeOH and MeNO_2_, the conversions obtained in MeNO_2_ were higher than those obtained for reactions carried out in MeOH as a solvent (Table 2). Takeya and co‐workers^[^ ^52^ ^]^ reported that for SnCl_4_‐mediated coupling reactions of 1‐naphthols, solvents with low donor numbers proved to be particularly favorable, which is a trend observed in the comparison between MeOH and MeNO_2_ in this reaction. However, higher yields and selectivities of **BNP** were obtained in MeOH than in MeNO_2_. The formation of multiple minor side products resulting from coupling reactions leads to low selectivities obtained in MeNO_2_ (Table 2). The reduction of the **BNP** yield in MeNO_2_ is due to the stabilization of intermediate species such as binaphthol (**BNL**), which was also observed in the oxidation of **MNL** on 5%Pt‐5%Bi/AC catalyst.^[^ ^6^ ^]^ **BNL** is the intermediate product toward the formation of **BNP**.^[^ ^22^ ^]^

It is very common to obtain high substrate conversion but with low product selectivities in reactions that are carried out at high temperatures due to side reactions competing with the formation of the target product.^[^ ^6^ ^]^ This was the case for the oxidation of **MNL** in refluxing MeOH (Table 2, entries 9‐12). There was an insignificant increase in **BNP** selectivity when the reaction was carried out under refluxing MeNO_2_ on MW‐5Au5Ru (Table 2, entries 13‐14). On the other hand, an increase in temperature in the oxidation of **TMHQ** led to a minimal increase in the selectivity of **TMBQ** (Table 2, entries 1‐8). A used MW‐5Au5Ru catalyst was tested for the oxidation of **TMHQ** under refluxing MeNO_2_ and 100% conversion of **TMHQ** was obtained with a 100% yield of **TMBQ** (Table 2, entry 9). This shows that this catalyst is stable and recyclable, and can be reused without or with minimal loss of its activity.

## 3 Conclusion

The structural properties of the catalysts vary according to their fabrication methods. Both MW‐5Au5Ru and DP‐5Au5Ru contain FCC Au and hexagonal phases of Ru metal in their bulk samples with ruthenium metal oxides on their surfaces (XRD and XPS studies). MW‐5Au5Ru has agglomerated Au particles with a high Ru metal dispersion and APS of 2.58 nm while DP‐5Au5Ru contains interlaced Au–Ru nanochains on the surface of the silica support. Furthermore, the catalyst preparation methods appear to influence the BET surface areas, as evidenced by the increase in the surface area of the DP‐5Au5Ru catalyst relative to the support, which is possibly attributable to the restructuring or formation of amorphous silica on the surface of the support during the catalyst preparation step. An increase in the surface area could also be possibly due to the isolated agglomerated Au–Ru nanochains present on this catalyst (SEM and TEM).

The oxidation of **TMHQ** and oxidative coupling of **MNL** were carried out over silica‐supported 5Au5Ru catalysts prepared by MW or DP, with H_2_O_2_ as an oxidant to yield **TMBQ** and **BNP** as products, respectively. Both MW‐5Au5Ru and DP‐5Au5Ru exhibited outstanding catalytic activities in the oxidation of **TMHQ**, in which **TMHQ** conversions of over 100% were achieved. However, MW‐5Au5Ru catalyst is a notable exception with **TMHQ** conversion of 58.2% in MeOH at RT. The catalytic activity data indicated that reactions in both MeOH and MeNO_2_ promote high **TMHQ** conversions, with MeNO_2_ providing slightly higher yields and selectivities of **TMBQ** than MeOH. The slight reduction of **TMBQ** selectivities in MeOH is due to the formation of byproducts, which seemed to be prominent in MeOH than in MeNO_2_. This may be due to the high donor number and polarity of MeOH. For the oxidation of **MNL**, low conversion of **MNL** and low yields of **BNP** at RT for both DP‐5Au5Ru and MW‐5Au5Ru in MeOH were obtained. Increasing the temperature from RT to 96 °C in MeNO_2_ increased **MNL** conversion but did not enhance the selectivity of the **BNP** significantly. In general, it can be concluded that DP‐5Au5Ru catalyst is more active than MW‐5Au5Ru catalyst. Furthermore, MeOH supports the formation of byproducts in comparison to MeNO_2_ in the reaction of **TMHQ**. However, the opposite was observed in the oxidative coupling of **MNL**. It can therefore be concluded that the interaction of the solvent with the substrate is one of the key influences in the catalytic outcome of the reaction.

In conclusion, the catalyst synthesis method, solvent, and reaction temperature (RT or reflux) have an influence on the outcome of the reaction. These parameters impact substrate conversion, as well as the yield and selectivity of the intended product. The silica‐supported 5Au5Ru catalysts utilized in this study are highly efficient for the production of **TMBQ** from **TMHQ**, as well as **BNP** from **MNL**. This catalytic approach is anticipated to play a significant part in creating more chemically viable and ecologically friendly strategies for the production of chemicals with economical value.

## Supporting Information

The materials that were used to support the findings of this study are given in the supporting information file.

## Conflict of Interest

The authors declare no conflict of interests.

## Supporting information

Supplementary Material

## Data Availability

The data that support the findings of this study are available in the supplementary material of this article.

## References

[1] M. F. McLaughlin , E. Massolo , S. Liu , J. S. Johnson , J. Am. Chem. Soc. 2019, 141, 2645. 30698429 10.1021/jacs.8b13006 PMC6411290

[2] A. A. Adeniyi , P. A. Ajibade , Bioinorg. Chem. 2018, 1, 5796287. 10.1155/2018/5796287 PMC6008838 29967635

[3] P. M. S. Sahoo , S. Behera , R. Behura , A. Acharya , D. Biswal , S. K. Suna , R. Sahoo , R. C. Soren , B. R. Jali , Biointerface Res. Appl. Chem. 2022, 12, 3247.

[4] N. V. G. Moorthy , S. V. Pansare , Tetrahedron 2018, 74, 142.

[5] A. Flores , E. Cots , J. Bergès , K. Muñiz , Adv. Synth. Catal. 2019, 361, 2.

[6] M. V. Maphoru , J. Heveling , S. Kesavan Pillai , Eur. J. Org. Chem. 2016, 2016, 331.

[7] D. V. Jawale , E. Gravel , V. Geertsen , H. Li , N. Shah , I. N. N. Namboothiri , E. Doris , ChemCatChem 2014, 6, 719.

[8] M. Eggersdorfer , D. Laudert , U. Létinois , T. McClymont , J. Medlock , T. Netscher , W. Bonrath , Angew. Chem. Int. Ed. 2012, 51, 12960. 10.1002/anie.201205886 23208776

[9] S. Ganapaty , P. S. Thomas , S. Fotso , H. Laatsch , Phytochem. 2004, 65, 1. 10.1016/j.phytochem.2004.03.011 15184011

[10] S. Wang , Y. Cheng , X. Wang , Q. Yang , W. Liu , J. Am. Chem. Soc. 2022, 144, 14945. 35943208 10.1021/jacs.2c06969

[11] L. M. Fombad , South Afr. J. Anaesth. Analg. 2023, 29, S158.

[12] S. Vahr , W. De Blok , N. Love‐Retinger , B. Thoft‐Jensen , B. Turner , G. Villa , J. Hrbácek , Intravesical instillation with mitomycin C or bacillus Calmette‐Guerin in non‐muscle invasive bladder cancer, https://nurses.uroweb.org, (accessed: August 2024).

[13] D. O. Futuro , P. G. Ferreira , C. D. Nicoletti , L. P. Borba‐Santos , F. C. D , a Silva , S. Rozental , V. F. Ferreira , Na. Acad. Bras. Ciên. 2018, 90, 1187. 10.1590/0001-3765201820170815 29873671

[14] J. Hassan , M. Sèvginon , C. Gozzi , E. Schulz , M. Lemaire , Chem. Rev. 2002, 102, 1359. 11996540 10.1021/cr000664r

[15] S. Yang , G. Xu , S. Shi , H. Xin , J. Gao , Z. An , Catal. Commun. 2019, 123, 105.

[16] O. A. Kholdeeva , I. D. Ivanchikova , O. V. Zalomaeva , A. B. Sorokin , I. Y. Skobelev , E. P. Talsi , J. Phys. Chem. B 2011, 115, 11971. 21913639 10.1021/jp2055975

[17] O. O. Uchewa , O. J. Ezugworie , J. Trace Elem , Med. Biol. 2019, 52, 192. 10.1016/j.jtemb.2018.12.016 30732882

[18] S. A. Baker‐Dockrey , A. L. Lukowski , M. R. Becker , A. R. Narayan , Nat. Chem. 2018, 10, 119. 29359749 10.1038/nchem.2879 PMC6503525

[19] T. A. Fassbach , J.‐M. Ji , A. J. Vorholt , W. Leitner , ACS Catal. 2024, 14, 7289.

[20] O. A. Kholdeeva , O. V. Zalomaeva , Coord. Chem. Rev. 2016, 306, 302.

[21] F. Derikvand , F. Bigi , R. Maggi , C. G. Piscopo , G. Sartori , J. Cat. 2010, 271, 99.

[22] M. V. Maphoru , J. Heveling , S. K. Pillai , ChemPlusChem 2014, 79, 99. 31986771 10.1002/cplu.201300307

[23] O. A. Kholdeeva , O. V. Zalomaeva , A. B. Sorokin , I. D. Ivanchikova , C. D. Pina , M. Rossi , Catal. Today 2007, 121, 58.

[24] M. V. Maphoru , J. Heveling , S. Kesavan Pillai , Kinet. Catal. 2017, 58, 441.

[25] M. V. Maphoru , J. Heveling , S. Kesavan Pillai , J. Catal. 2017, 348, 47.

[26] T. Lekgetho , L. F. Mabena , M. Tukulula , M. V. Maphoru , ChemistrySelect 2024, 9, e202402857.

[27] M. V. Maphoru , J. Heveling , S. Kesavan Pillai , React. Kinet. Mech. Catal. 2021, 134, 95.

[28] S. Swain , A. M. Antony , S. A. Patil , A. K. Sama , Mater. Today Nano. 2023, 24, 100416.

[29] A. Jouve , G. Nagy , F. Somodi , C. Tiozzo , A. Villa , A. Balerna , A. Beck , C. Evangelisti , L. Prati , J. Catal. 2021, 368, 324.

[30] Y. H. Ke , X. Wang , J. Y. Li , H. Li , H. Yuan , Russ. J. Phys. Chem. A 2021, 95, 264.

[31] P. Jin , X. Liang , Y. He , S. Liu , X. Zhu , Eur. J. Inorg. Chem. 2014, 25, 4144.

[32] M. Hävecker , P. Düngen , S. Buller , A. Knop‐Gericke , A. Trunschke , R. Schlögl , Catal. Struct. React. 2017, 3, 104.

[33] Z. Wu , E. Borretto , J. Medlock , W. Bonrath , G. Cravotto , ChemCatChem 2014, 6, 2762.

[34] A. G. R. Howe , R. Maunder , D. J. Morgan , J. K. Edwards , Catalysts 2019, 9, 748.

[35] J. M. Campelo , D. Luna , R. Luque , J. M. Marinas , A. A. Romero , ChemSusChem 2009, 2, 18. 19142903 10.1002/cssc.200800227

[36] E. A. Paoli , F. Masini , R. Frydendal , D. Deiana , C. Schlaup , M. Malizia , T. W. Hansen , S. Horch , I. E. Stephens , I. Chorkendorff , Chem. Sci. 2015, 6, 190. 28553467 10.1039/c4sc02685c PMC5424673

[37] W. Wang , S. Guo , I. Lee , K. Ahmed , J. Zhong , Z. Favors , F. Zaera , M. Ozkan , C. S. Ozkan , Sci. Rep. 2014, 4, 4452. 24663242 10.1038/srep04452 PMC3964521

[38] S. Maikap , W. Banerjee , T. C. Tien , T. Y. Wang , J. R. Yang , J. Nanomater. 2011, 2011, 810879.

[39] W. P. Griffith , The Chemistry Of Rarer Platinum Metals (Eds: F. A. Cotton , G. Wilkinson ), Interscience Publishers, New York 1967, p. 491.

[40] X. Chen , Z. Jia , Z. Liu , X. Wang , M. Liang , RSC Adv. 2023, 13, 3255. 36756428 10.1039/d2ra07650k PMC9890632

[41] https://xpsdatabase.com , (accessed: August 2024).

[42] I. Maack , M. Osmić , L. Mohrhusen , P. Buhani , K. Al‐Shamery , ChemNanoMat 2021, 7, 658.

[43] O. V. Zalomaeva , N. N. Trukhan , I. D. Ivanchikova , A. A. Panchenko , E. Roduner , E. P. Talsi , A. B. Sorokin , V. A. Rogov , O. A. Kholdeeva , J. Mol. Catal. A: Chem. 2007, 277, 185.

[44] T. Ogata , I. Okamoto , H. Doi , E. Kotani , T. Takeya , Tetrahedron Lett. 2003, 44, 2041.

[45] T. Takeya , H. Doi , T. Ogata , I. Okamoto , E. Kotani , Tetrahedron 2004, 60, 9049.

[46] G. Strukul , F. Somma , N. Ballarini , F. Cavani , A. Frattini , S. Guidetti , D. Morselli , Appl. Catal. A: Gen. 2009, 356, 162.

[47] Y. Cheneviere , V. Caps , A. Tuel , Appl. Catal. A 2010, 387, 129.

[48] D. T. Sawyer , J. L. Roberts , Experimental Electrochemistry for Chemists, John Wiley & Sons, Inc, NY 1974.

[49] S. Okumu , J. Chem. 2024, 3, 24.

[50] V. Gutmann , Electrochim. Acta 1976, 21, 661.

[51] V. Gutmann , The Donor‐Acceptor Approach to Molecular Interactions, Plenum Press, New York 1978, p. 595.

[52] T. Takeya , H. Doi , T. Ogata , T. Otsuka , L. Okamoto , E. Kotani , Tetrahedron 2004, 60, 6295.

