# Supplementary material for: Effects of Catalyst Synthesis Methods on the Physicochemical Properties of Silica‐Supported Au–Ru Bimetallic Catalysts and their Influence on the Oxidation of Phenols with H2O2
Source: ChemistryOpen. 2025 Apr 10;14(6):e202400484. doi: 10.1002/open.202400484 (PMC12138045; doi:10.1002/open.202400484)
Supplement: Supplementary file 1 — Supplementary Material [file OPEN-14-e202400484-s001.pdf]

Effects of Catalyst Synthesis Methods on the Physicochemical Properties of Silica-Supported Au-Ru Bimetallic Catalysts and their Influence on the Oxidation of phenols with H_2O_2

Tumisang Lekgetho,[a] Matshawandile Tukulula,[b] Letlhogonolo F Mabena,[a] and Mabuatsela V Maphoru*[a]

Supporting materials

Experimental

SM 1. Catalyst preparation

The catalyst preparation methods were previously described by us.[26] All catalyst precursors, solvents, support and substrates used in this work did not undergo any purification process prior their application.

Microwave-assisted loading (MW): The preparation of 5%Au-5%Ru/SiO_2 (MW-5Au5Ru) was carried out by adding tetrachloroauric (III) acid (0.0861 g, 0.2186 mmol) and ruthenium (III) chloride (207.43 g/mol, 0.1024 g, 0.4947 mmol) into a 250 mL flask containing acetone-H_2O mixture (50%v/v, 50 mL). The mixture was subjected to a constant ultrasonication to dissolve the salts. The silica (0.9001 g) was added to the mixture. The mixture was ultrasonicated for 30 min. The mixture was transferred into a Teflon vessel and irradiated in the Anton Paar Multiwave PRO Microwave at a power of 600 W and temperature of 190°C (Ramp time to 190°C: 5 min, and irradiation time at 190°C: 10 min). The solid sample was filtered and washed 5 times with deionized water and dried in an oven at 120°C for 24 h.

Deposition method (DP): The 5%Au-5%Ru/SiO_2 (DP-5Au5Ru) catalyst was synthesised in a 250 mL round-bottom flask by dissolving tetrachloroauric (III) acid (0.0864 g, 0.2194 mmol) and ruthenium (III) chloride (0.1025 g, 0.4947 mmol) in acetone-H_2O mixture [50%v/v, 50 mL]. The mixture was subsequently ultrasonicated for 10 min to dissolve the salts. The silica (0.9000g) was introduced into the aqueous solution of the mixed precursors while agitating the mixture with a magnetic stirring bar. A reductant solution, $NaBH_4$ (1.0 M, 1.5 mL, 1.5 mmol), was added dropwise to the mixture to reduce the metal ions under constant ultrasonic agitation for 30 min. The mixture was further stirred with a magnetic stirring bar for 10 min. The solid sample was obtained through a vacuum filtration and washed under vacuum with deionized water. The sample was oven-dried at 120°C for 24 h.

SM 2. Catalyst characterization

Surface textures of the catalysts were characterized by Micromeritics Tristar II 3020 3.02 surface area and porosity analyzer. Prior to nitrogen physisorption analysis, about 0.05 g of the catalyst was degassed for 3 h at 100°C. This was followed by nitrogen physisorption analysis for 5 h at -196°C (77 K) in the pressure range of 0.866 kPa to 87.1 kPa. Physisorption data were obtained using the BET, BJH and t-plot methods. Metal loadings on the catalysts were determined by using SpectroArcos FHS ICP-OES instrument. Crystallographic structures of the catalysts were determined on Bruker D8 Advance diffractometer operating at 40 kV voltage, 40 mA current and a scan speed of 0.1 s/step at r.t using a Ni-filtered Cu K_α radiation at λ = 0.15418 nm and 2θ range of 5-90°. Crystallographic phases were identified with the aid of Bruker DIFFRACplus evaluation software (EVA) in conjunction with the Crystallography Open Database (COD) for powder diffractions. A ZEISS-Auriga Cobra field-emission scanning electron microscope with a focused-ion beam and energy dispersive X-ray spectrometer was used for examining the morphology and elemental composition of the catalysts. Carbon was used to sputter-coat the specimens prior the analysis. A 5.00 kV electron accelerating voltage was selected for capturing the images while 15.0 kV was used for EDX analysis. A JEOL JEM-2100 High resolution transmission electron microscope (HRTEM) with an electron acceleration voltage of 200 kV was used for capturing the images of the catalysts. The catalysts were dispersed through ultrasonication in EtOH for 1 min followed by deposition of the catalyst onto the carbon-coated copper grids (200 mesh size). ImageJ software was used to determine the particle sizes of the NPs. Surface elemental compositions and chemical states of the catalysts were assessed on Thermo Scientific ESCAlab 250Xi system equipped with a monochromatic Al kα radiation source (hv = 1486,7 eV). The measurements were carried out with a base pressure of <10-8 mBar, X-ray power of 300 W and X-ray spot size of 900 μm. The survey spectra were captured with a dwell time of 9 min 3.9 s, binding energy (BE) range extending from 0 to 1350 eV, pass energy of 100 eV and step size of 1 eV. High-resolution spectra were acquired with a pass energy of 20 eV. The data was processed and compared with the reference spectra from the International XPS database. The FT-IR spectra scans from 4000 to 450 cm^{-1} were generated by using a Perkin Elmer Spectrum Two Spectrophotometer fitted with two universal attenuated total reflectance Fourier transform infrared spectrometers, KBr windows, and a $LiTaO_3$ detector. The catalysts samples were placed on the diamond, and a force gauge of 130-150 N was applied onto the samples for analysis. The ^{1}H NMR and ^{13}C NMR spectra were obtained from 400/54/ASP Varian 400 MHz premium-shielded NMR spectrometer. The organic products were dissolved in deuterated chloroform prior the analysis.

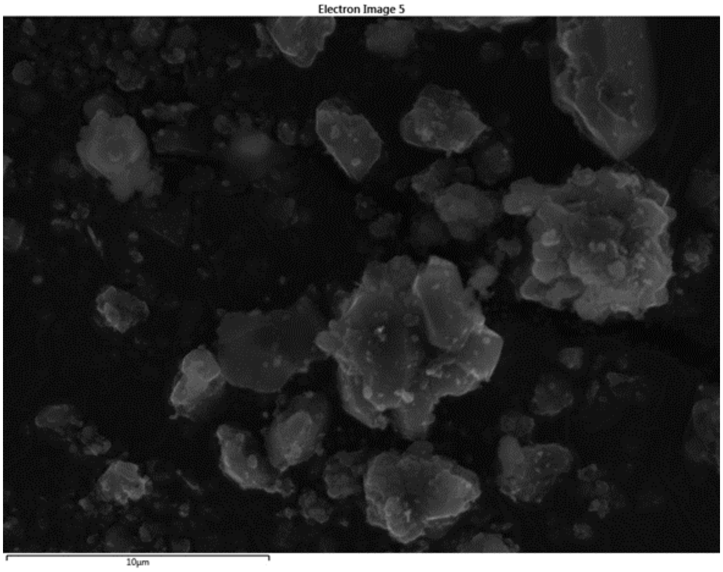

SM 3. EDX map image for MW-5Au5Ru on Figure 4

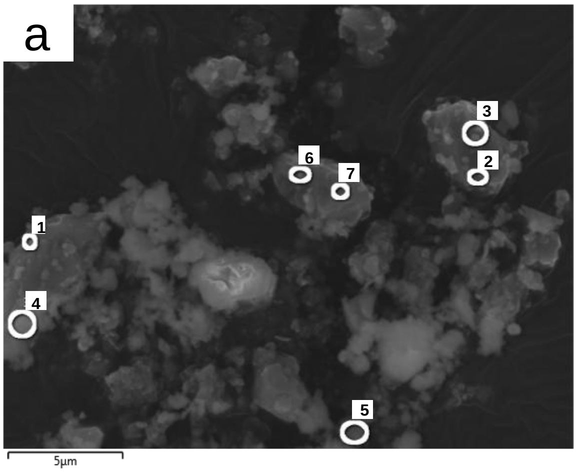

Point	Si (%)	O (%)	Au (%)	Ru (%)
1	10.3	28.1	-	-
2	27.6	47.1	-	-
3	34.1	46.6	-	-
4	15.5	39.2	0.8	0.2
5	16.4	35.2	-	0.4
6	33.0	50.1	-	-
7	37.0	51.1	-	-

SM 4. EDX “point-and-shoot” analysis of MW-5Au5Ru

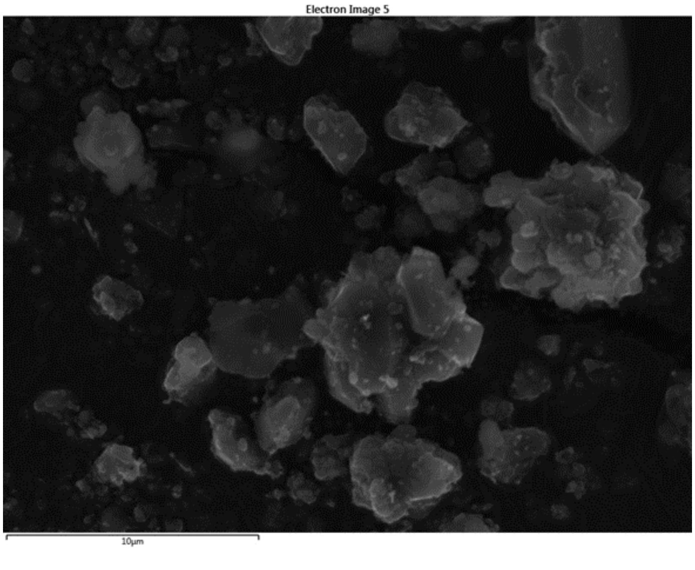

SM 5. EDX map image of DP-5Au5Ru on Figure 5b

SM 6. Oxidation reactions and product separation

Catalyst testing was carried out as previously reported by us. [26] A **TMHQ** (3.2854 mmol) and catalyst (0.0300 g) were added into a 250 mL two-neck round-bottom flask. A solvent (20 mL) was added to the mixture and agitated to dissolve **TMHQ** using a magnetic stirring bar. An aqueous H_2O_2 (30%, 3.0 mL, 22.0 mmol H_2O_2) was constantly added to the mixture over 2 h through a peristaltic pump under constant magnetic stirring at r.t. After the reaction, the catalyst was separated from the reaction mixture through vacuum filtration and washed with MeOH. The solvent was stripped off from the product by using a rotary evaporator. The component of the residual liquid was analysed on the UV-active TLC plate using **TMHQ** as a reference. The products were separated using a silica column chromatography. The reactions conducted under solvent refluxing temperatures were carried out in a two-neck round-bottom flask fitted with a condenser and the peristaltic pump inlet. The oil bath was used to heat the reaction mixture. The same procedures were followed for the oxidation of **MNL** (2.8703 mmol) with a reaction time of 1 h. The structural characterization of the products, **TMBQ** and **BNP**, were previously reported by us.[22,26]
